# Impact of an HIV Prevention Intervention on Condom Use among Long Distance Truckers in India

**DOI:** 10.1007/s10461-012-0314-y

**Published:** 2012-09-25

**Authors:** Sachin Juneja, Vasudha Rao Tirumalasetti, Ram Manohar Mishra, Shekhar Sethu, Indra Ramyash Singh

**Affiliations:** 1 Transport Corporation of India Foundation, Gurgaon, India; 2 HIV & AIDS Program, Population Council, 142, First Floor, Golf Link, New Delhi, 110003 India; 3 Family Health International, New Delhi, India

**Keywords:** Truckers, Intervention, Propensity scores, HIV

## Abstract

This paper examines the impact of three components of an HIV prevention program (mid-media, interpersonal communication, and project-run clinics) on consistent condom use by long distance truckers with paid and non-paid female partners in India. Data from 2,723 long distance truckers were analyzed using the propensity score matching approach. Based on utilization of services, the following categories of intervention exposure were derived: no exposure, exposure only to mid-media, exposure only to mid-media and interpersonal communication, exposure only to mid-media and project-run clinics, and exposure to all three intervention components. Compared to those who were not exposed to any intervention, exposure to mid-media alone increased consistent condom use with paid female partners by about ten percent. Exposure to mid-media and visits to project-run clinics increased consistent condom use with non-paid female partners by 26 %. These findings suggest that mid-media events and clinics were the most effective package of services to increase consistent condom use among the long distance truckers.

## Introduction

Long distance truck drivers and their helpers (hereafter referred to as truckers) are considered to be at high risk for sexually transmitted infections (STI) and HIV in many parts of the world, including Asia [1–4], Africa [5–8], South America [9, 10] and the United States [11, 12]. Truckers’ increased HIV risk is largely attributed to long periods of absence from the family [11, 13, 14].

In India, recent evidence shows that around one-fourth of truckers have sex with female sex workers (FSWs) [15] and less than three-fourth use condoms consistently in sex with these partners [14–16]. Substantial proportions of truckers also have sexual relations with non-paid female partners [14–16] and only about one-third use condoms consistently in such sexual encounters [15, 16]. Studies in the country have found high HIV prevalence (2–16 %) [14, 15, 17, 18] and high STI prevalence (3–13 %) among truckers [15, 17, 18].

Due to high risk behaviours, high STI and HIV prevalence, and ability to transmit infections to new geographic areas, HIV prevention interventions among truckers started in early 1990s with aim to raise the awareness through group discussion on matters related to HIV, using posters and pamphlets with positive AIDS messages, street theatres and puppetry, and distribution of condoms [19–21]. Some interventions also used one-to-one and one-to-group education sessions through peers, and provided treatment for STI at truckers’ halt points [22, 23]. The HIV prevention efforts among truckers were strengthened towards the end of 1990s when a large-scale HIV prevention program among truckers, named as ‘Healthy Highway’ was initiated. The project offered services including treatment and counseling for STIs, condom promotion, dissemination of educational materials, and face-to-face behavioural change communication to truckers at major halt points where at least 50 trucks were parked at any time of the day or night [24]. In 1998, the second phase of the National AIDS Control Program initiated specific interventions among truckers that were largely based on the strategies of the Healthy Highway program [25]. The Healthy Highway program was handed over to the government in 2000 which replaced the central management team of the Healthy Highway program by independent State AIDS Control Societies [26].However, despite several individual HIV prevention programs for truckers being implemented through State AIDS Control Societies, the need for a large-scale intensive program was recognized due to lack of coordination among interventions within or across states, language barriers, and the absence of a standard network of services to address the needs of truckers [26]. In this context, a large-scale intervention, called Kavach (meaning ‘shield’ in Hindi/Sanskrit) for truckers in India was started as part of Avahan, the India AIDS initiative [27]. In addition to working with truckers, Avahan supported interventions among other high risk groups such as FSWs and their clients, men who have sex with men/transgenders and injecting drug users in several high HIV prevalence states of India [27, 28]. This paper describes the Kavach intervention and examines the impact of its components on condom use behavior by truckers with non-regular female sexual partners.

## Kavach Intervention

The Kavach intervention was initiated in 2004 in 36 transshipment locations across India covering the major national highways along six routes, namely, North–East, North–West, North–South, West–South, South–East and West–East. Transshipment locations are places where transporters and brokers operate to link truckers with clients who need to transport goods, and routes are road corridors on which truckers travel.

The Kavach intervention was redesigned in 2006 by halving the number of implementation sites from 36 to 17 to focus on the major truck halt points in nine Indian states. Unlike the other HIV prevention programs to cover large number of intervention sites for truckers coverage, the redesigned program aimed to provide maximum coverage to truckers by working with high intensity program (i.e., ensuring more contacts by peer educators, greater program visibility and increased number of project-run clinics) at major transshipment locations in the country where large number of truckers halt and wait for their consignments.

The Kavach intervention had three main components: mid-media events; peer-led dialogue based interpersonal communication (IPC) and project-run clinics. Mid-media events included street plays, health games, film shows, truckers’ festivals and the distribution of audio cassettes/compact discs. Mid-media events aimed to communicate messages on safe sex practices to truckers and motivate them to visit project-run clinics. Trained professionals conducted about ten street plays every month at each intervention site. Health games were conducted twice a day and film shows were organized once a week at each intervention site. Truckers’ festivals were large-scale events conducted annually to create an interest among brokers, transporters and truckers in the program. Audio cassettes/compact discs with catchy local songs interspersed with spoofs on popular film actors delivering HIV prevention messages and endorsing services at project-run clinics were distributed to truckers so as to reinforce positive messages on HIV when they travel on the highway. The project monitoring data indicate that nationally around 150,000 audio cassettes/compact discs were distributed every year.

In the Kavach intervention, IPC included one-to-group discussions by trained peer educators who were either active truckers or ex-truckers. About 10–12 IPC sessions were conducted every day at each intervention site. In order to reinforce messages already received in the mid-media events, those messages were repeated during the IPC sessions.

One static clinic and two mobile clinics were established at each intervention site to provide services at the door-step of truckers’ halting points. To avoid becoming stigmatized as STI clinics, the project clinics treated a range of general health ailments in addition to providing counseling services by a qualified counselor on safe sex practices, correct and consistent condom use, and diagnosis and treatment of STI so that truckers would not hesitate to visit these clinics. Following the McDonald’s business franchisee model, where the look and service of outlets do not differ across any location [29], project-run clinics were branded with uniform appearance and services across all intervention sites to facilitate greater acceptance and easy recognition by truckers. Social marketing of condoms was promoted in partnership with condom marketing organizations by opening non-traditional outlets such as at tea stalls, and installing condom vending machines at strategically chosen places in the implementation sites. Details of the Kavach intervention are available elsewhere [26].

## Methods

### Study Setting and Sampling

Of the 17 intervention sites, six were purposively chosen as survey sites for data collection. The selected sites were located at Ghaziabad and Delhi in northern India, Indore in central India, Mumbai and Pune in western India, and Bengaluru in southern India. These sites were chosen because truckers from different parts of the country wait at these places for a considerable length of time for new consignments. Data were collected during February–August 2009 in two phases at each selected site in order to cover wider group of truckers’ population. A total sample size of 2,800 truckers was estimated for the survey.

The total sample size was distributed across the six transshipment locations chosen for the survey in proportion to the number of truckers available at those transshipment locations. Parking areas for trucks were considered as clusters in each transshipment location for the multistage cluster sampling and all clusters were selected for the survey. The registration number of the trucks parked in each cluster on the day of the survey was listed and a systematic random sampling approach was used to select the trucks. The main driver or helper of the selected trucks was approached to participate in the survey.

Respondents were selected only if they met the following three criteria: (i) aged 18 years or more; (2) had worked as a trucker for the past 2 years or more; and (3) were working in a truck with a national permit (i.e., permission from the government to carry goods to and from any part of the country). Truckers who had already participated in the survey (either in an earlier phase of the survey or in the same phase) were not interviewed again.

A pre-coded questionnaire translated into the local language was used to collect data by trained native speakers. A total of 2,810 truckers were interviewed in the survey. Of these, cases who were traveling on the South–East route [30]; cases exposed only to IPC [20] and exposed only to project-run clinics [31]; and cases where there was incomplete information on exposure to intervention [2] were excluded from the analyses due to the small number of observations. This resulted in a total analytical sample of 2,723 cases.

### Ethical Considerations

A comprehensive informed consent process was followed. Respondents were informed about the study including the duration of the interview (approx. 30 min), and their queries addressed before written consent was taken. To protect confidentiality all questionnaires were entirely anonymous and the names and addresses of respondents were not recorded. To maintain privacy, interviews were either conducted inside the truck or in a secluded public area such a corner road where others would not be able to listen to the interview. Participants were not given any monetary compensation for their time in the study.

### Measures

#### Socio-Demographic Characteristics

The socio-demographic characteristics considered in this paper were age (completed years), formal schooling (no, yes), marital status (not currently married, currently married), usual place of residence (native place, others), route generally travelled (North–East, North–South, North–West, West–South and West–East), duration of work as a trucker (completed years), occupation (driver, helper) and availability of music player in the truck (no, yes). Formal schooling was defined as the ability to both read and write.

#### Exposure to the Intervention

Exposure to mid-media was defined as exposure to at least one mid-media events (watched street plays, participated in health games, watched film shows, participated in truckers’ festivals, received audio cassettes/compact discs) in the past 12 months (no, yes). Exposure to IPC was defined as participation in at least one IPC session in the past 12 months (no, yes). Exposure to project-run clinics was defined as at least one visit to a project clinic (either static or mobile) in the past 12 months (no, yes). Exposure to any intervention was defined as exposure to at least one of the three intervention components, namely, mid-media, IPC and project-run clinics in past 12 months.

To measure exposure to different intervention components in the past 12 months, a variable with the following five mutually exclusive categories were derived: no exposure, exposure only to mid-media, exposure only to mid-media and IPC, exposure only to mid-media and project-run clinics, and exposure to all three intervention components.

#### Consistent Condom Use

Consistent condom use with paid female partners and non-paid female partners was the primary outcome variable measuring HIV-related risk behaviour. A paid female partner was defined as a woman to whom the respondent had paid cash in exchange for sex. A non-paid female partner was defined as a woman to whom the respondent was not married and did not pay cash in exchange for sex. Consistent condom use with a sexual partner was defined as condom use in every sexual encounter with that particular partner (no, yes).

### Statistical Analyses

Basic descriptive statistics (i.e., proportions, means and standard deviations) were presented to describe participants’ socio-demographic characteristics, exposure to the intervention, and outcome indicators. The bivariate association between the categories of intervention exposure and outcome indicators was examined using χ^2^ test.

The propensity score matching (PSM) approach was adopted to examine the impact of truckers’ exposure to any intervention as well as exposure to different categories of intervention on consistent condom use by them with paid and non-paid female partners. Studies indicate that when only observational data are available without a valid control group, the PSM approach can be adopted to evaluate the impact of intervention exposure on an outcome [30–34]. The PSM approach to examine the impact of intervention exposure on consistent condom use included two steps. First, for each respondent a propensity score was calculated using multivariate logistic regression, with variable measuring exposure to intervention as the binary dependent measure and observed socio-demographic characteristics as covariates. The next step consisted of matching exposed and unexposed truckers with similar propensity scores.

The observed socio-demographic characteristics including age, literacy, marital status, occupation, duration of work as trucker, time away from family, and route category were considered as the covariates because these characteristics have been found to correlate with truckers’ sexual risk behaviors in India [11, 14, 17, 35, 36]. We added presence of music player in the truck as one of the covariates as it was likely to affect truckers’ exposure to mid-media intervention components (distribution of audio-cassettes/compact discs).

Matching was carried out using the radius method in which each exposed trucker was matched only with the unexposed truckers whose propensity score fell into a predefined neighborhood of the estimated propensity score of the exposed trucker [37]. The predefined neighborhood was determined by estimating the radius as one-fourth of the standard deviation of the corresponding propensity scores [37–39]. The common support restriction (to exclude data from exposed truckers with a propensity score higher than that of any unexposed trucker) was imposed to improve the quality of the matches [30]. Among unexposed truckers, those who had propensity scores similar to exposed truckers were termed as matched unexposed and they served as controls for the exposed group. Matching was done with replacement so that one matched unexposed (or exposed) trucker could potentially serve as match for several exposed (or unexposed) truckers [37].

The key assumption in the PSM approach is that conditional on the propensity score, assignments to exposed and unexposed groups can be considered as random [32]. One test of this assumption is to examine the balancing property which states that, conditional on the propensity scores, the distribution of confounding factors are similar among exposed and matched unexposed groups [38]. To examine whether the balancing property was satisfied, differences in the socio-demographic characteristics of exposed truckers with all unexposed truckers (to assess the differences before matching) and matched unexposed truckers (to assess the differences after matching) were tested for significance using the χ^2^ test (for percentages) and unpaired *t* test (for mean values). The overall covariate imbalance of the model was examined by testing the joint significance of all the regressors (ability of covariates to predict exposure to any intervention) using the likelihood ratio test before and after matching.

The PSM approach enabled us to estimate the average effect of intervention exposure among exposed truckers (difference in condom use among exposed truckers and matched unexposed truckers) and the average effect of intervention exposure among unexposed truckers (difference in condom use of unexposed truckers and matched exposed truckers). The average effect of intervention exposure among exposed truckers measured the impact of intervention on exposed truckers whereas the average effect of intervention exposure among unexposed truckers measured the impact that the interventions would have had on unexposed truckers if they were exposed. These two effects were weighted by the proportion of truckers exposed and unexposed respectively to arrive at the impact of intervention exposure on consistent condom use which measured the increase in consistent condom use due to intervention exposure [30, 32]. As a final diagnostic check, sensitivity analyses were conducted to assess the potential effect of unmeasured confounders on the results. The Rosenbaum bounds approach was adopted, which is described elsewhere [32].

Following these steps, we first examined the impact of exposure to any intervention on condom use by comparing the consistent condom use among truckers exposed to any intervention with that among matched unexposed truckers. Since exposure to mid-media was common among all the exposed truckers, categories with significant impact on outcomes were examined against exposure only to mid-media for assessing the additional impact different components. Analyses were conducted using the statistical software STATA (version 11).

## Results

### Socio-Demographic Characteristics, Exposure to Intervention, and Sexual Behavior

Mean age of the respondents was about 30 years. Of the 2,723 respondents, more than three-fourth (83.4 %) had formal schooling, nearly two-third (66.6 %) were currently married, and about one-fifth (22.1 %) were working as helpers. A total of 991 (36.4 %) truckers were not exposed to the intervention in the past 12 months, 651 (23.9 %) were exposed only to mid-media, 188 (6.9 %) were exposed only to mid-media and IPC, 449 (16.5 %) were exposed only to mid-media and clinics, and 444 (16.3 %) were exposed to all three intervention components. About 45 % of respondents reported sex with paid female partners in the past 12 months and almost three-fourths (74.1 %) reported using condoms consistently in such sexual encounters. About 22 % of reported having sex with non-paid female partners and less than half (41.8 %) had used condom consistently in such sexual acts (Table 1).

**Table 1:** Socio-demographic characteristics, intervention exposure, and sexual behavior among truckers, India, 2009 (*N* = 2,723)

Socio-demographic characteristics, intervention exposure, and sexual behavior	Percentage and mean
Socio-demographic characteristics	
Mean age in years (SD)	30.2 (8.0)
Formal schooling^a^	83.4
Marital status	
Currently married	66.7
Not currently married^b^	33.3
Occupation	
Driver	77.9
Helper	22.1
Mean duration of working as trucker in years (SD)	8.4 (6.5)
Route categories	
North–East	20.1
North–South	20.6
North–West	31.2
West–South	11.8
West–East	16.2
Living at native place	56.6
Intervention Exposure	
No exposure	36.4
Exposed only to mid-media	23.9
Exposed only to mid-media and IPC	6.9
Exposed only to mid-media and clinics	16.5
Exposed to all three intervention components	16.3
Exposed to any intervention^c^	63.6
Sexual behavior	
Sex with paid female partners in the past 12 months	44.6
Consistent condom use with paid female partners^d^	74.1
Sex with non-paid female partners in the past 12 months	21.8
Consistent condom use with non-paid female partners^e^	41.8

*SD* standard deviation, *IPC* interpersonal communication

^a^Formal schooling refers to the ability to both read and write

^b^Not currently married includes truckers who were never married, divorced or widower

^c^Exposure to any intervention was defined as exposure to at least one of the three intervention components, namely, mid-media, IPC and project-run clinics in the past 12 months

^d^Among those who reported sex with paid female partners in the past 12 months

^e^Among those who reported sex with non-paid female partners in the past 12 months

### Intervention Exposure and Consistent Condom Use

Consistent condom use with both paid and non-paid female partners was significantly higher among truckers exposed to any intervention than those with no intervention exposure (paid female partners: 76.5 % vs 65.8 %, χ^2^ statistic = 12.85, *p* < 0.001; non-paid female partners: 45.3 % vs 32.5 %, χ^2^ statistic = 7.82, *p* = 0.005) (Table 2). Consistent condom use with paid female partners varied significantly from about two-thirds (63.3 %) among truckers exposed only to mid-media to more than three-fourth (80.7 %) among those exposed only to mid-media and project-run clinics (χ^2^ statistic = 23.56, *p* < 0.001). Consistent condom use with non-paid female partners also varied from about one-third (31.8 %) among truckers exposed only to mid-media and IPC, to almost half among those exposed to mid-media and project-run clinics (54.9 %) and those exposed to all three intervention components (50.9 %) (χ^2^ statistic = 24.67, *p* < 0.001).

**Table 2:** Truckers’ sexual behavior by exposure to intervention components, India, 2009 (*N* = 2,723)

	Exposure to any intervention^a^			Categories of intervention exposure				
Sexual behavior in past 12 months	No (*N* = 991)	Yes (*N* = 1,732)	*p* value^b^ (test Statistic)	Only mid-media (N = 651)	Only mid-media and IPC (N = 188)	Only mid-media and project-run clinic (N = 449)	All three intervention components (N = 444)	*p* value^c^ (test Statistic)
Sex with paid female partners (%)	28.3	53.3	<0.001 (166.92)	43.3	47.6	61.4	64.5	<0.001 (229.91)
Sex with non-paid female partners (%)	16.1	25.0	<0.001 (29.23)	16.4	27.5	24.9	36.6	<0.001 (93.29)
Consistent condom use with paid female partners^d^ (%)	65.8	76.5	<0.001 (12.85)	77.3	63.3	80.7	76.0	<0.001 (23.56)
Consistent condom use with non-paid female partners^e^ (%)	32.5	45.3	0.005 (7.82)	31.8	34.6	54.9	50.9	<0.001 (24.67)

*IPC* interpersonal communication

^a^Exposure to any intervention was defined as exposure to at least one of the three intervention components, namely, mid-media, IPC and project-run clinics in the past 12 months

^b^ *p* values are obtained by comparing percentages for those who were exposed to any intervention and those who were not exposed to any intervention using Person’s χ^2^ test statistic. Estimated values of the χ^2^ statistic are given in brackets below the corresponding *p*-values

^c^ *p* values are obtained by comparing percentages across categories of intervention exposure using χ^2^ statistic. Estimated values of the χ^2^ statistic are given in brackets below the corresponding *p* values

^d^Among those who reported having sex with paid female partners in the past 12 months

^e^Among those who reported having sex with non-paid female partners in the past 12 months

### Matching of Exposed and Unexposed Truckers

Among truckers who had sex with paid female partners, those exposed to any intervention differed significantly from all unexposed truckers in terms of almost all the socio-demographic characteristics (Table 3). However, no significant differences in socio-demographic characteristics were found when truckers exposed to any intervention were compared with matched unexposed truckers. Similar results were observed for truckers who reported sex with non-paid female partners. The likelihood ratio Chi-square statistic computed to test the joint insignificance of covariates reduced significantly after matching among truckers reporting sex with paid female partners (before matching: χ^2^ statistic = 99.6, *p* < 0.001; after matching: χ^2^ statistic = 4.6, *p* = 0.948) as well as among those reporting sex with non-paid female partners (before matching: χ^2^ statistic = 74.4, *p* < 0.001; after matching: χ^2^ statistic = 11.3, *p* = 0.418). Similar results were obtained while comparing socio-demographic characteristics of all unexposed and matched unexposed truckers with the socio-demographic characteristics of truckers exposed to different categories of intervention exposure (results not shown in tabular form but available upon request).

**Table 3:** Matching socio-demographic characteristics of truckers who had sex with paid and non-paid female partners in the past 12 months, India, 2009

	Had sex with paid female partners					Had sex with non-paid female partners				
Socio-demographic Characteristics	Exposed to any intervention^a^	All unexposed	*p* value^b^ (test statistic)	Matched unexposed	*p* value^c^ (test statistic)	Exposed to any intervention^a^	All unexposed	*p* value^b^ (test statistic)	Matched unexposed	*p* value^c^ (test statistic)
Average age (years)	30.7	29.0	0.001 (3.26)	30.5	0.900 (0.13)	29.0	26.2	<0.001 (4.13)	29.1	0.605 (0.52)
Formal schooling^d^ (%)	84.7	78.3	0.012 (2.51)	87.6	0.066 (1.84)	87.5	78.8	0.008 (2.68)	89.1	0.447 (0.76)
Currently married (%)	68.4	57.3	0.001 (3.48)	68.2	0.987 (0.02)	59.4	38.8	<0.001 (4.54)	58.8	0.976 (0.03)
Working as driver (%)	85.4	68.3	<0.001 (6.57)	85.9	0.717 (0.36)	82.9	58.1	<0.001 (6.50)	83.9	0.641 (0.47)
Average duration of work as a trucker (years)	8.5	7.4	0.008 (2.65)	8.3	0.923 (0.10)	7.9	5.8	<0.001 (4.00)	7.6	0.836 (0.21)
Have music player in truck (%)	83.3	73.3	<0.001 (3.75)	82.7	0.802 (0.25)	79.9	67.5	0.002 (3.19)	77.1	0.379 (0.88)
Traveling on North–East route (%)	15.8	21.0	0.040 (2.05)	16.9	0.560 (0.58)	10.2	17.5	0.015 (2.44)	6.9	0.076 (1.78)
Traveling on North–South route (%)	23.8	16.7	0.012 (2.51)	21.9	0.401 (0.84)	19.9	16.3	0.319 (1.03)	23.7	0.128 (1.52)
Traveling on North–West route (%)	33.5	35.2	0.602 (0.52)	34.2	0.969 (0.04)	34.4	31.3	0.470 (0.72)	37.0	0.452 (0.75)
Traveling on West–South route (%)	15.4	9.6	0.014 (2.47)	14.6	0.856 (0.18)	18.9	10.6	0.016 (2.42)	15.1	0.136 (1.49)
Traveling on West–East route (%)	11.5	17.4	0.009 (2.63)	12.4	0.575 (0.56)	16.6	24.4	0.032 (2.15)	17.3	0.852 (0.19)
Living at native place (%)	62.9	45.9	<0.001 (5.13)	60.9	0.481 (0.70)	64.9	51.9	0.004 (2.91)	61.2	0.315 (1.01)
N^e^	933	281		277		433	160		157	
Likelihood ratio χ^2^ test statistic (*p* value)^f^										
Before matching	99.6 (<0.001)					74.4 (<0.001)				
After matching	4.6 (0.948)					11.3 (0.418)				

^a^Exposure to any intervention was defined as exposure to at least one of the three intervention components, namely, mid-media, IPC and project-run clinics in the past 12 months

^b^ *p* values were obtained by comparing percentages for those who were exposed to any intervention and all unexposed truckers. All unexposed refer to truckers who were not exposed to any intervention. Differences in percentages were tested using χ^2^ test statistic and differences in average values were tested using unpaired *t* test statistic. Estimated values of the test statistic are given in brackets below the corresponding *p* values

^c^ *p* values were obtained by comparing percentages for those who were exposed to any intervention and matched unexposed truckers. Matched unexposed refers to truckers who were unexposed and had propensity scores similar to that for those exposed group. Differences in percentages were tested using χ^2^ test statistic and differences in average values were tested using unpaired *t* test statistic. Estimated values of the test statistic are given in brackets below the corresponding *p* values

^d^Formal schooling refers to the ability to both read and write

^e^Differences in *N* values for matched unexposed and all unexposed indicate number of unexposed cases dropped from the analysis as no match could be found for them

^f^The Likelihood Ratio χ^2^ test statistic was used to test the joint significance of all the regressors (that is, the ability of covariates to predict exposure to any intervention) before and after matching. The estimated value of the χ^2^ test statistic and corresponding *p* values (in brackets) are shown

### Impact of Intervention Exposure on Consistent Condom Use

Table 4 shows the impact of truckers’ exposure to different intervention components on consistent condom use with paid and non-paid female partners. When compared with no exposure, the exposure to any intervention increased consistent condom use with paid female partners by 7.2 % (95 % confidence interval (CI): 4.5–16.9, *p* = 0.001) and consistent condom use with non-paid female partners by 12.4 % (95 % CI: 4.3–21.2, *p* = 0.003). Exposure to mid-media alone increased consistent condom use with paid female partners by 9.9 % (95 % CI; 4.0–18.9, *p* = 0.003) as compared with no exposure; however, this was not found to have an impact on consistent condom use with non-paid female partners. Similarly, exposure to only mid-media and project-run clinics increased consistent condom use with paid female partners by 11.4 % (95 % CI: 8.2–21.5, *p* < 0.001) and consistent condom use with non-paid female partners by 26.0 % (95 % CI: 11.6–33.3, *p* < 0.001) when compared with no exposure.

**Table 4:** Percent increase in condom use among truckers with paid and non-paid female partners in the past 12 months by exposure to intervention components, India, 2009

	Consistent condom use with paid female partners				Consistent condom use with non- paid female partners			
Exposure to intervention components	Average effect of exposure among exposed^a^ (%)	Average effect of exposure among unexposed^b^ (%)	Expected increase in consistent condom use^c^ (%)	95 % CI for expected increase in consistent condom use	Average effect of exposure among exposed^a^ (%)	Average effect of exposure among unexposed^b^ (%)	Expected increase in consistent condom use^c^ (%)	95 % CI for expected increase in consistent condom use
Any intervention^d^ versus no intervention	6.1	10.7	7.2	(4.5–16.9)**	12.4	10.8	12.4	(4.3–21.2)**
Only mid-media versus no intervention	9.6	10.2	9.9	(4.0–18.9)**	1.5	−3.7	−1.5	(-7.1–5.2)
Only mid-media and IPC versus no intervention	−0.2	−4.7	−3.5	(−6.1–8.3)	−0.1	−0.8	−0.7	(-6.9–9.1)
Only mid-media and clinic versus no intervention	10.5	12.3	11.4	(8.2–21.5)***	28.5	24.1	26.0	(11.6–33.3)***
All three intervention components versus no intervention	4.1	9.4	6.6	(4.7–18.2)*	19.7	7.8	11.6	(6.1–30.8)**
Only mid-media and clinic versus only mid-media	5.2	-0.9	2.1	(−3.9–10.7)	17.4	21.8	19.6	(11.1–35.2)***
All three intervention components versus only mid-media	−1.4	−4.9	−3.1	(−8.4–5.7)	7.6	10.6	8.8	(7.7–30.6)**

*CI* confidence intervals, *IPC* interpersonal communication

*p < 0.05; **p < 0.01; ***p < 0.001

^a^Average effect of exposure among exposed truckers measured the impact of intervention on exposed truckers

^b^Average effect of exposure among unexposed truckers measured the impact that the interventions would have had on unexposed truckers if they were exposed

^c^It represents the impact of the program obtained by averaging the impact across all the individuals (exposed and unexposed)

^d^Exposure to any intervention was defined as exposure to at least one of the three intervention components, namely, mid-media, IPC and project-run clinics in past 12 months

The additional impact of project-run clinics on consistent condom use with paid female partners was not evident when compared with exposure to only mid-media. However, visits to project-run clinics increased consistent condom use with non-paid female partners by about 20 % (95 % CI: 11.1–35.2, *p* < 0.001) as compared to exposure to mid-media alone. Similar results about the additional combined impact of IPC and project-run clinics were found while comparing exposure to all three components with exposure to mid-media alone.

### Sensitivity Analyses

The estimated impact of truckers’ exposure to any intervention on consistent condom use with paid female partners was insensitive to a bias introduced by unobserved confounders that increase the odds of exposure to any intervention up to 30 % (Mantel–Haenszel statistic = 1.85, *p* = 0.030) (Table 5). Similarly, its impact on consistent condom use with non-paid female partners remained insensitive to the presence of an unobserved confounder that increased the odds of exposure up to 20 % (Mantel–Haenszel statistic = 1.71, *p* = 0.044). Similar results were obtained for estimated impact of exposure to different categories of intervention exposure on consistent condom use with paid and non-paid female partners.

**Table 5:** Sensitivity analysis: Rosenbaum bounds for the estimated impact of exposure to intervention on consistent condom use with paid and non-paid female partners, India, 2009

Exposure to intervention components	Consistent condom use with paid female partners		Consistent condom use with non-paid female partners	
	Permissible odds of differential exposure to intervention due to unobserved factors	*p* value^a^ (test statistic)	Permissible odds of differential exposure to intervention due to unobserved factors	*p* value^a^ (test statistic)
Any intervention^b^ versus no intervention	30 %	0.032 (1.85)	20 %	0.044 (1.71)
Only mid-media versus no intervention	30 %	0.048 (1.66)	NA	NA
Only mid-media and project-run clinic versus no intervention	50 %	0.029 (1.89)	60 %	0.038 (1.77)
All three intervention components versus no intervention	20 %	0.034 (1.82)	30 %	0.041 (1.74)
Only mid-media and clinic versus only mid-media	NA	NA	50 %	0.033 (1.82)
All three intervention components versus only mid-media	NA	NA	30 %	0.044 (1.73)

NA: The sensitivity analyses were not done as exposure to the corresponding components of intervention did not result in a significant difference in condom use with paid female partners and non-paid female partners

^a^ *p* values were estimated using Mantel–Haenszel statistic under the assumption of overestimation of the impact of exposure to corresponding component of intervention. Estimated values of Mantel–Haenszel test statistic are given in brackets below the corresponding *p* values

^b^Exposure to any intervention was defined as exposure to at least one of the three intervention components, namely, mid-media, IPC and project-run clinics in the past 12 months

## Discussion

The study findings showed differential effects of exposure to components of the Kavach intervention on consistent condom use with paid and non-paid female partners. For instance, trucker’s exposure to mid-media alone as compared to those who were not exposed to any intervention reported significantly higher consistent condom use with paid female partners. Whereas, truckers who visited project-run clinics as compared to those with exposure to mid-media alone have reported higher consistent condom use with non-paid female partners. Exposure only to mid-media and IPC, without being supplemented by visits to project-run clinics, did not have a significant impact on condom use behavior.

The finding that truckers’ exposure to mid-media alone has increased consistent condom use with paid female partners can be explained, to some extent, by the possible confounding effect of parallel intensive interventions among FSWs in several states of India [15]. The national AIDS control program as well as the Avahan supported initiatives for the social marketing of condoms at ‘hotspots’ in high HIV prevalence states in India have increased the availability of condoms in high risk settings where commercial sex takes place [28, 40]. In the context of several intervention programs to promote condom use in commercial sex, information on the availability and importance of condom use imparted through the mid-media could lead to high rates of consistent condom use with paid female partners. However, as the study findings indicate, exposure to mid-media alone did not increase consistent condom use with non-paid female partners, which may perhaps be explained by two factors. First, the degree of intimacy and trust is higher with non-paid female partners than paid partners, and hence condom use with such partners is low [14–16]. Second, non-commercial sex takes place in settings (e.g., homes) where the reach and effectiveness of HIV prevention programs may be limited.

Information on safe sexual practices provided by mid-media events coupled with counseling by a qualified counselor on safe sexual practices, correct and consistent condom use and treatment of STI by a qualified doctor in project-run clinics appear to be the most effective package of services to increase condom use with both paid and non-paid female partners. Findings suggest that visits to project-run clinics did not have an additional impact as compared to exposure to mid-media alone in terms of consistent condom use with paid female partners. However, visits to project-run clinics had an additional impact on consistent condom use with non-paid female partners compared to exposure to mid-media alone. One possible reason behind success of project-run clinics in increasing consistent condom use with non-paid female partners could be the in-depth one-to-one discussions with qualified counselors and qualified doctors. Research from developing countries indicate that HIV counseling recipients are more likely to use condom than those who do not receive it [41]. A recent study among cross-border truck drivers in Hong Kong also found voluntary HIV counseling and testing plus the information dissemination approach to be more effective in promoting safe sex behaviors than the information dissemination approach alone [42]. Although further studies are needed to understand the processes that motivate truckers to use condoms with non-paid female partners, this paper suggests that the provision of clinics with integrated one-to-one counseling by trained professionals promotes consistent condom use, particularly with non-paid female partners.

Findings also indicate that as compared to no exposure, exposure to only mid-media and IPC did not improve condom use behavior. This could be due to the small sample size of truckers exposed to these two intervention components, as well as the fact that truckers who were exposed to only mid-media and IPC constitute a subgroup who did not visit the project-run clinics even after being exposed to two intervention components that were aimed to motivate them to visit these clinics. As this group of truckers could not be convinced to visit clinics even after repeated efforts, it may be argued that these truckers did not give much importance to the messages on condom use provided during mid-media events and IPC sessions. Hence, though covered by the program, these truckers’ condom use behavior was similar to those who were not exposed to any intervention component. These findings suggest the need to change the IPC approach (e.g., using photographs to illustrate the consequences of STI) to motivate truckers to use condoms with non-regular female sexual partners.

The percentage of truckers exposed to the intervention as observed in this study was found to be higher than that found in a large-scale survey among long distance truck drivers conducted during 2009–10 in India [15]. This may be because our study was limited to transshipment locations where the Kavach intervention program was being implemented whereas the above-mentioned study included transshipment locations where this program did not operate.

On the other hand, the levels of consistent condom use with paid female partners (74.1 %) and non-paid female partners (41.8 %) as found in this study are consistent with other large-scale study among long distance truckers in India which found that about 74 % of the truck drivers used condoms consistently with paid female partners whereas about 37 % of them used condom consistently with non-paid female partners [15]. In recent past, small-scale studies in different parts of the country have found a wide range (57 %–84 %) of estimates for consistent condom use by truckers with paid female partners. This wide variation in estimates across the small-scale studies are often due to small sample size, and variation in geographic region. Differentials in sexual behaviors of truckers from different parts of the country have been documented elsewhere [15, 35].

This study has some limitations, which should be considered when interpreting the results. First, findings are based on self- reports, which are vulnerable to social desirability and recall biases. The survey measured a limited number of socio-demographic characteristics and did not include some of the important variables such as income and alcohol consumption, which may confound the results. Finally, although the PSM approach was used to establish a causal relationship between exposure to intervention and consistent condom use, this should not be considered an alternative approach to randomized trials with valid case and control groups.

In conclusion, the study documents that the Kavach intervention resulted in a significant increase in consistent condom use with both paid and non-paid female partners. Exposure to mid-media alone substantially increased consistent condom use by truckers with paid female partners whereas exposure to project-run clinics had a significant impact on increasing condom use with non-paid female partners. These findings suggest the need for intensive mid-media events to increase awareness of HIV and easily accessible clinics that provide counseling on safe sex practices and STI treatment services to truckers to increase the adoption of consistent condom use with non-regular female partners. It also highlights the need to understand truckers’ risk perceptions, needs and the barriers to the uptake of project-run clinic services even after repeated attempts to promote behavior change.
